# Revisiting suicide prevention in later life: mixed-methods study protocol

**DOI:** 10.1192/bjo.2026.12011

**Published:** 2026-05-22

**Authors:** M. Isabela Troya, Paul Corcoran, Derek Chambers, Anne M. Doherty, Donagh Hennebry, Katerina Kavalidou, Caoimhe Lonergan, Sally Ann Lovejoy, Faraz Mughal, Michael Norton, Emma Wallace, Ella Arensman

**Affiliations:** School of Public Health, University College Cork, Ireland; National Suicide Research Foundation, Cork, Ireland; National Mental Health, Health Service Executive Access and Integration, Cork, Ireland; Psychiatry, School of Medicine, University College Dublin, Ireland; Liaison Psychiatry, Mater Misericordiae University Hospital, Dublin, Ireland; Resource Office for Suicide Prevention, Health Service Executive South West Mental Health Services, Killarney, Ireland; Department of Psychiatry, University College Cork, Ireland; National Clinical Programme for Self-Harm and Suicide-related Ideation, Dublin, Ireland; School of Medicine, Keele University, UK; Oxford Primary Care Clinical Trials Unit, University of Oxford, UK; Adult Continuing Education, University College Cork, Ireland; Department of General Practice, School of Medicine, University College Cork, Ireland

**Keywords:** Self-harm, suicide, older adults, mixed methods, coroner

## Abstract

**Background:**

Globally, suicide is more prevalent in older adults compared with any other age group. Although some research has identified risk and protective factors for suicidal behaviour in older adults, further research is needed to provide an up-to-date overview to inform service delivery.

**Aims:**

This study protocol describes mixed-methods research that will examine and identify factors associated with self-harm and suicide in older adults (aged 60 years and older) living in Ireland.

**Method:**

Four stages will be conducted. First, data from the National Self-Harm Registry of Ireland (NSHRI), will be used to examine recent hospital self-harm presentations of older adults, including during the COVID-19 pandemic. Second, a case-series study will examine the adverse life events and psychosocial factors experienced by older adults before dying by suicide, using data from closed coronial files, through the Irish Probable Suicide Deaths Study (IPSDS). Third, risk of suicide following hospital-presenting self-harm will be examined among older adults via data linkage of the NSHRI and IPSDS. Finally, using semi-structured interviews, the service needs of older adults with suicidal behaviour will be explored from the perspectives of older adults, carers and healthcare practitioners.

**Conclusions:**

A comprehensive understanding of adverse events and psychosocial factors associated with the suicidal behaviour of older adults is needed to inform service provision. This proposed research is aligned with (inter)national priorities, mental health promotion and suicide reduction policies. It aims to address gaps in mental healthcare interventions for older adults at risk of suicide.

Suicide prevention is a worldwide priority. Every year, over 746 000 people die by suicide globally,^1^ with 14.6 million people affected by self-harm annually.^2^ In this article, we use the self-harm definition as described in Box 1, which includes the broad range of behaviours from non-suicidal self-injury to attempted suicide.^3^ Although suicide and self-harm are not the same within the suicidal crisis spectrum, self-harm is the strongest predictive risk factor for suicide.^4^ In 2021, global suicide rates were estimated at 9.0 per 100 000 for all age groups, with older adults aged 70 and older having the highest mortality rates (males: 37.9 per 100 000, females: 15.6 per 100 000).^1,5^ The total number of hospital-presenting self-harm episodes globally is unknown, because of limited self-harm surveillance data; however, it has been estimated that self-harm episodes are 62.5 per 100 000.^1^ Internationally, across all age groups, men more often die by suicide (12.8 per 100 00) compared with women (5.4 per 100 000); however, as per the gender paradox^6^ self-harm is most prevalent among females (74 per 100 000) compared with males (51 per 100 000).^1^ Although older adults have the highest suicide rates globally,^1,5^ this trend varies across regions.

Box 1 Self-harm definition.

We used the definition of self-harm given by Platt et al, as ‘an act with non-fatal outcome in which an individual deliberately initiates a non-habitual behavior, that without intervention from others will cause self-harm, or deliberately ingests a substance in excess of the prescribed or generally recognized therapeutic dosage, and which is aimed at realizing changes that the person desires via the actual or expected physical consequences’.^3^

Older adults have distinct patterns of suicidal behaviour, including higher suicidal intent, greater lethality and lower help-seeking.^7^ Furthermore, physical illness, social isolation and bereavement are commonly reported factors to suicidal behaviour in older adults.^8^ Despite suicide rates being highest in older adults globally, self-harm in older adults is generally less prevalent.^7,9^ A systematic review estimated varying rates of self-harm, from 19 to 65 per 100 000.^10^ This rate was calculated from evidence of 40 articles and over 62 000 older adults; however, most evidence was from English-speaking and high-income countries, limiting the generalisability of results. Other studies have calculated rates of self-harm in older adults, estimating a mean annual rate from 2003 to 2016 of 83.8 per 100 000 person years in England,^11^ 37.4 per 100 000 in 2022 in Australia^12^ and 57.8 per 100 000 in Ireland between 2007 and 2019.^13^ Recent research conducted in Ireland found that in the second year (2021) following the start of the COVID-19 pandemic, significant increases were observed in suicide-related hospital presentations in older adults.^14^ The COVID-19 pandemic increased social isolation, health fears and reduced access to health services.^15^ Older adults were one of the groups mostly affected by the pandemic, given their clinical vulnerability to the virus, which resulted in several countries, including Ireland, implementing stay-at-home orders for older adults, among other vulnerable groups. No research has examined hospital-presenting self-harm rates during the past 5 years in Ireland, which limits our understanding of the potential impact of the pandemic to older adults’ mental health.

Identification of risk factors for repeated self-harm and suicide is key to inform suicide prevention. Individual and social determinants have been linked to self-harm in older adults.^7^ Older adults with previous psychiatric history, including depression, living alone and living with comorbid physical health conditions, are at increased risk of self-harm.^8,10^ In England, research found that older adults who self-harm are 67 times more likely to die by suicide.^16^ National research from Australia^17^ and Ireland^18^ reported higher suicide risk among individuals of all ages who had previously presented to hospital with self-harm. In these studies, suicide was associated with male gender and older age.^17,18^ Healthcare professionals supporting these older adults are aware of the increased risk of suicide following self-harm.^7,19,20^

In Ireland, suicide prevention is a key priority, with the current National Suicide Prevention Strategy having as one of its seven goals, ‘to improve surveillance, evaluation and high-quality research relating to suicidal behaviour’.^21^ Furthermore, Ireland has been described as one of the fastest ageing populations in the European Union, with 15% of the population aged 65 years or older.^22^ Internationally, mental health promotion and suicide prevention among older people is a global priority, as evidenced by the World Health Organization’s Euro Mental Health Coalition.^23^ National research to date has outlined hospital self-harm trends among older adults up to 2019,^13,24^ and has highlighted the elevated suicide risk among individuals presenting with self-harm, with older age identified as a key risk factor.^18^ However, the clinical and sociodemographic characteristics of older adults who die by suicide remain unknown, as well as specific risk factors for suicide in this age group following hospital-presenting self-harm. Furthermore, no qualitative research has explored the health service needs of Irish older adults experiencing suicidal behaviour.

## Study aim and research questions

The overall aim of this research is to improve understanding of the factors associated with self-harm and suicide risk in older adults living in Ireland. This study will address the following research questions:

- What was the immediate and medium-term impact of the COVID-19 pandemic and associated public health measures on older adult hospital self-harm presentations? Were there any demographic subgroups of older adults that were at increased risk of hospital-presenting self-harm?
- What psychosocial factors are associated with suicide among older adults?
- What psychosocial factors are associated with suicide risk following hospital-presenting self-harm in older adults?
- What are the psychosocial circumstances behind self-harm in older adults, and what are the service needs of older adults experiencing suicidal behaviour from the perspectives of older adults, carers and healthcare practitioners supporting these older adults?

## Method

### Research design and setting

This protocol describes a mixed-methods study with concurrent design that involves four stages of research. The proposed research will use multiple data sources and methods to address its objectives, and a concurrent triangulation mixed-methods design guided by the overall study aim to integrate qualitative and quantitative data at an analysis and interpretation phase.^25,26^ Integrating quantitative and qualitative methods will allow for convergence of data to address the research objectives. The study has not yet started.

### Stage 1. Investigating the effects of the COVID-19 pandemic on self-harm rates in older adults in Ireland

#### Study design and population

Secondary analysis will be conducted with a national cohort of hospital-presenting self-harm among older adults living in Ireland.

Ireland is one of the few countries worldwide that has a national surveillance system for self-harm as recommended by the World Health Organization.^27,28^ The National Self-Harm Registry Ireland (NSHRI) has full national coverage of all public hospitals in Ireland and has been recording emergency department hospital presentations of self-harm nationally for over 20 years.

All presentations made by older adults aged 60 years and older who presented to emergency departments in Ireland following self-harm between 1 January 2007 and 31 December 2024, will be included using the NSHRI. The following variables, routinely collected by the NSHRI, will be examined: gender, age, type of residence (household, hospital, prison, homeless, other), name and quantity of drugs involved if intentional drug overdose, date and hour of hospital presentation, method(s) of self-harm, alcohol involvement, urban/rural residence (Dublin city, other cities and towns), receipt of mental health assessment and recommended next care following treatment in the emergency department.

The outcome variable will be hospital self-harm presentations in older adults aged 60 years and older.

#### Statistical analysis

Time-series analysis^29,30^ will be conducted to examine trends in monthly self-harm incidence among older adults aged 60 years and older residing in Ireland between January 2019 and December 2024. Aggregated count data on hospital-presenting self-harm will be obtained from the NSHRI. Monthly incidence rates will be calculated per 100 000 population by using data from the Central Statistics Office (CSO). The 2022 census will be used for that year, whereas CSO national mid-year estimates will be used for 2019–2021 and 2024. Incident counts will be stratified by gender and age group (60–69, 70–79 and 80 years and older). We will examine the feasibility of using 5-year age-band stratification during the analysis phase, and will retain these categories where case numbers are sufficient; if cell sizes are too small to allow meaningful interpretation, 10-year age groups will be used instead.

**Fig. 1:**
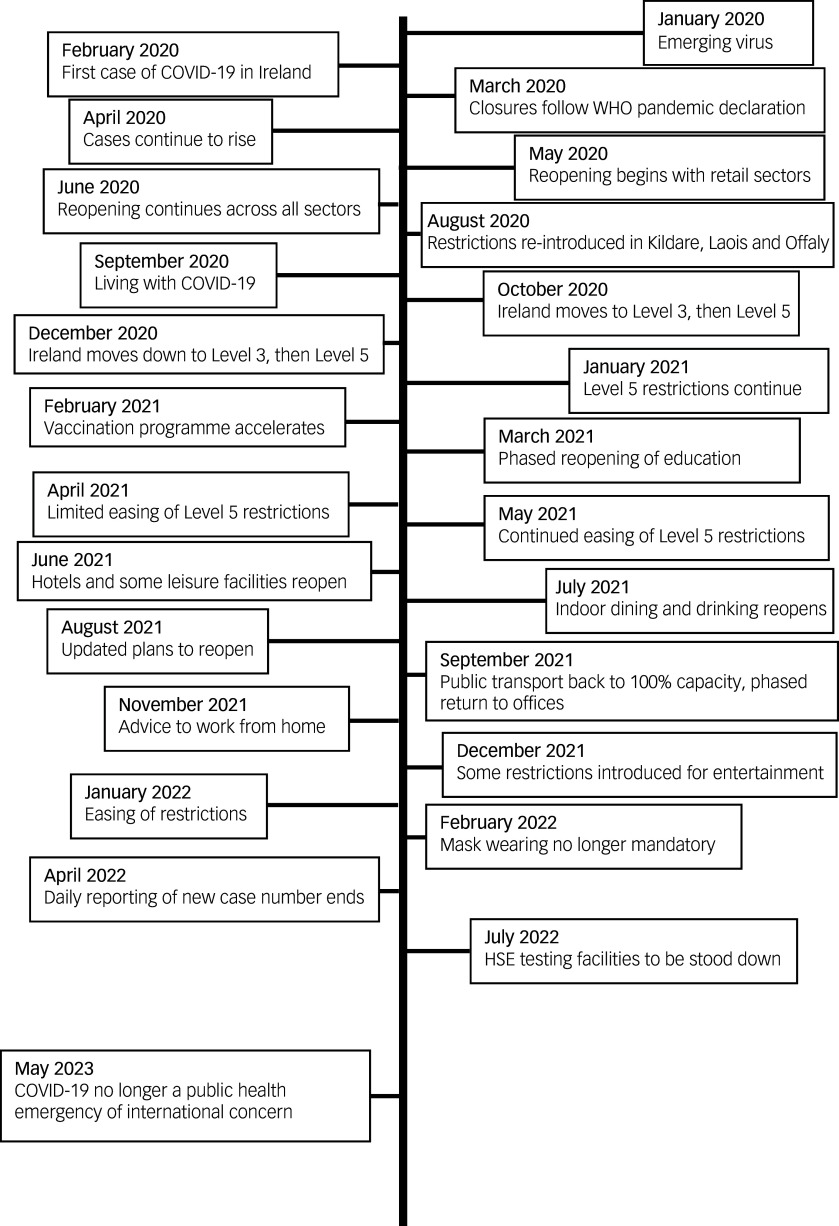
Visual timeline of COVID-19 events in Ireland, adapted from the Central Statistics Office.^31^ WHO, World Health Organization; HSE, Health Service Executive.

To allow for analysis of changes in rates over the observation period, the 72-month data-set will be divided into four time periods that correspond to different social restrictions in place during the COVID-19 pandemic. The time periods were selected according to public health guidance provided by the National Public Health Emergency Team and Department of Health Ireland (see Fig. 1) and described in Table 1.

**Table 1:** Time periods for the analysis of Work Package 1

Time period	Social restrictions information
Pre-pandemic: 1 January 2019 to 29 February 2020	The first case of COVID-19 in Ireland was confirmed in February 2020 and social restrictions started in March 2020
Pandemic phase 1: 1 March 2020 to 31 December 2021	Varying levels of social restrictions were in place, including social distancing, stay-at-home orders and mandatory face masks. Older adults were advised to ‘cocoon’ or social distance
Pandemic phase 2: 1 January 2022 to 31 December 2022	Most social restrictions were removed in January 2022 (including ‘cocooning’ orders for older adults), with mask mandates removed in March 2022
Post-pandemic: 1 January 2023 to 31 December 2024	Although SARS-CoV-2 was still present within the population, all social restrictions relating to COVID-19 were lifted

Negative binomial regression models will be used to account for overdispersion in the count data. Incidence rate ratios and 95% confidence intervals will be reported to quantify differences between time periods and reported by gender and age group (60–69, 70–79 and 80 years and older). Incidence rate ratios will be estimated to compare the rates during the pandemic phase 1, pandemic phase 2 and post-pandemic periods, using the pre-pandemic period as the reference group. Stata version 19 for Windows (StataCorp, Texas, USA; https://www.stata.com/) software will be used.

Given the availability of national self-harm data since 2007, longer-term time-series analysis may also be conducted. Interrupted time-series regression may be used to estimate changes in the level and slope of self-harm rates and joinpoint regression may be used to detect the timing of changes in trend with particular focus on the onset of the pandemic in March 2020.

#### Power considerations

In 2019, there were approximately 950 000 older adults aged 60 years and older living in Ireland. Based on previous research conducted using the NSHRI,^13^ in 2019 there were approximately 605 older adults aged 60 years and older presenting to hospital with self-harm, indicating a rate of 63.7 per 100 000. Crude Poisson or negative binomial regression models will detect a 12% change (rate ratio of 0.88 or 1.12) in this reference rate at *p* < 0.05.

### Stage 2. Identifying psychosocial and clinical circumstances behind suicide in older adults in Ireland

#### Study design and population

Retrospective case series of all suicide deaths in older adults aged 60 years and older living in Ireland. Adults aged 18–59 years will be used as a comparison group.

#### Data source

The Irish Probable Suicide Deaths Study (IPSDS) is a 6-year rich data-set that includes coroner-determined and research-determined suicides, nationally, based on information from closed coronial files (2015–2020). The IPSDS includes sociodemographic, clinical and adverse life events that preceded a suicide death, based on all relevant information that coroners gather during a death investigation. This national database was commissioned by the Health Service Executive in response to obtaining further information and detail to suicides, compared with the basic descriptive suicide mortality data, which can be accessed in the CSO. The IPSDS follows the standardised data collection methodology of the National Drug-Related Deaths Index.^32^

#### Outcome

The outcome will be death by suicide. The IPSDS defines suicide as ‘all deaths with a coronial suicide verdict (which, by definition, are considered to be suicide ‘beyond reasonable doubt’); and deaths that are more likely than not, based on the weight of evidence, to have been a suicide (research determined suicide/on the balance of probabilities)’.^32^ All probable suicides included in the IPSDS will be called suicides hereafter.

#### Measures

The IPSDS includes sociodemographic and clinical variables in the database. These are:

- Sociodemographic characteristics: gender, age, relationship status, living arrangements, parental status and socioeconomic group.
- Clinical risk characteristics: history of mental health condition, prescribed mental health medication, alcohol and drug use history, prior self-harm (e.g. suicide attempts that have required medically treatment or not), contact with health services and adverse life events.
- Death detail: Coroner’s verdict (beyond reasonable doubt/on the balance of probabilities), method of death, place of incident and suicide note left.

#### Statistical analysis

Descriptive statistics will be used to summarise all suicide deaths recorded in the IPSDS from 2015 to 2020. Frequencies and percentages will be calculated for all sociodemographic, clinical and death-related variables, stratified by age group (older adults aged 60 years and older versus adults aged 18–59 years) and by gender. Differences in categorical variables between age groups will be tested with the chi-squared test, or Fisher’s exact test where expected cell counts are small (i.e. <5). A *p*-value of <0.05 will be considered statistically significant.

To contextualise the extent of suicide among older adults in Ireland, crude suicide incidence rates per 100 000 population will be calculated for the study period (2015–2020), overall and stratified by age group (older adults versus adults) and gender. For older adults, 10-year age groups will be further calculated (60–69 years, 70–79 years, 80 years and older). We will examine the feasibility of using 5-year age-band stratification for older adults during the analysis phase, and will retain these categories where case numbers are sufficient; if cell sizes are too small to allow meaningful interpretation, broader 10-year age groups will be used instead. Further age-band stratification for the adult age group (18–59 years) is possible but outside the scope of this study; it may be explored in future analyses. Annual rates will also be estimated. The numerator will be all suicides recorded in the IPSDS, and the denominator will be national mid-year population estimates from the CSO for 2015 and 2017–2020. For 2016, CSO 2016 census data will be used. Exact Poisson 95% confidence intervals will be calculated for each rate. Analyses will be conducted with Stata version 19.

#### Power considerations

Suicide is a rare outcome, with approximately 100 older adults aged 60 years and older dying by suicide in 2017.^32^ In 2017, there were approximately 892 000 older adults aged 60 years and older living in Ireland, indicating an annual rate of 11 per 100 000. Based on a 6-year study period, crude Poisson or negative binomial regression models will detect differences of at least 18% (rate ratio of 0.82 or 1.18) between two groups of equal size at *p* < 0.05.

### Stage 3. Suicide risk following hospital presenting self-harm in older adults in Ireland

#### Study design and population

Prospective cohort study with retrospective data linkage of hospital-self-harm presentations of older adults aged 60 years and older, between January 2015 and December 2020 (the latest year for available data of the IPSDS). As a comparison group, we will examine adults aged 18–59 years during the same time period.

#### Exposure

The exposure of interest is a hospital-presenting self-harm event, as recorded in the NSHRI. Characteristics of the index self-harm episode (e.g. method used, provision of psychosocial assessment, region), and demographic variables (age, gender) will be included as covariates. Age group (18–59 *v*. 60 years and older) will serve as a key variable in comparative analyses.

#### Outcome

The primary outcome is death by suicide as defined by the IPSDS identified via linkage with the IPSDS. Individuals will be followed from the date of their index self-harm presentation until date of death by suicide or end of follow up period (31 December 2020).

The secondary outcome is self-harm repetition, defined as any subsequent hospital presentation for self-harm recorded in the NSHRI following the index episode. Time to first repeat event will be calculated, and multiple repetition episodes will be captured.

#### Measures

Variables examined will include demographic (gender, age, region), self-harm characteristics (method of self-harm, number of previous episodes, types of drugs taken) and clinical factors (provision of biopsychosocial assessment, next care recommendations offered by the hospital), all routinely collected as part of the NSHRI.

#### Data linkage process

A unique patient identifier has not yet been implemented in Ireland. The NSHRI has developed an algorithm to create a code based on personal identifiers and demographic data that identifies repeat presentations to hospital by the same individuals, without revealing identifying data on the individuals. The NSHRI code-generating application will be provided to the colleagues in the Health Research Board (HRB) who were responsible for the original collection of the IPSDS data. To facilitate data linkage the IPSDS anonymised and de-identifiable identifiers will be sent from the National Office of Suicide Prevention to the HRB. The HRB colleagues will run the application which will add the code to the IPSDS data. Using secure data transfer methods, the HRB colleagues will send the processed IPSDS data to am NSHRI researcher and co-author (P.C.). The NSHRI colleague will perform the data linkage based on matching NSHRI and IPSDS records with the same code. The NSHRI colleague will fully anonymise the linked data file by replacing the code with an arbitrary code, and will then provide the anonymised file to the first author. A similar process was employed for a similar study using data from the NSHRI and IPSDS.^18^

#### Statistical analysis

Descriptive statistics will be used to summarise the cohort by demographic and self-harm characteristics at baseline. To compare suicide risk following self-harm, crude suicide rates per 100 000 population for men, women and all persons will be calculated by age group (older adults aged 60 years and older versus adults aged 18–59 years) for both the self-harm cohort and the general population. For the general population, suicide cases without a history of hospital-presenting self-harm will be used as the numerator, and annual population estimates from the CSO will serve as the denominator. Exact Poisson 95% confidence intervals will be provided for these rates.

Poisson regression models will estimate the 12-month risk of suicide following the most recent episode of self-harm, compared with the general population, stratified by gender and age group. Individuals with a follow-up period of less than 12 months (i.e. those who present to hospital between 1 January 2020 and 31 December 2020) will be excluded from these analyses.

Kaplan–Meier survival curves will be generated to estimate time to suicide and repeat self-harm. Differences between age groups and other covariates (e.g. gender, method of self-harm) will be assessed with the log-rank test. To estimate the risk of repeat self-harm and suicide following self-harm, Cox proportional hazards regression models will be used to calculate hazard ratios and 95% confidence intervals, comparing older adults with adults aged 18–59 years. Models will be adjusted for relevant predictors/confounders (e.g. gender, method of self-harm, psychosocial assessment received). Age group will also be included as an effect modifier to assess whether risk patterns differ by age.

Proportional hazards assumptions will be tested. If assumptions are violated, alternative approaches such as Poisson regression or flexible parametric survival models will be considered. A similar Cox regression approach will be applied to assess risk factors for repeat self-harm. All analyses will be conducted with Stata version 19.

#### Power calculations

Based on previous NSHRI data,^13^ it is expected that approximately 600 individuals aged 60 years and older present to hospital annually with self-harm. Over 6 years (2015–2020), this yields a cohort of approximately 3600 individuals. Given estimated suicide rates in older adults following self-harm (ranging from 0.5 to 2% within 1–2 years),^18^ this sample is expected to provide sufficient power to detect meaningful hazard ratios (>2.0) for key predictors (e.g. gender, method of self-harm), assuming a significance level of 0.05 and 80% power.

### Stage 4. Examining the service needs of older adults with suicidal behaviour

#### Study design

A qualitative methods approach will be used. In line with the overall mixed-methods approach of the research, the theoretical approach underpinning this study design is pragmatism, which states that knowledge is constructed based on interactions between people and their environments.^33,34^ The perspectives, experiences and beliefs of older adults with previous suicidal behaviour, carers and healthcare professionals with experience of supporting older individuals who self-harm, will be gathered using semi-structured interviews. Pragmatism will allow for the understanding of participants’ perspectives and experiences with suicidal behaviour in older adults, acknowledging the unique context of each participant group.

#### Sampling and recruitment

Three participant groups will take part in this study, therefore sampling and recruitment for each of these groups is detailed separately. Exclusion criteria for all participant groups: not being able to provide consent, not being fluent in the English language and not living in Ireland.

#### Older adults

Older adults aged 60 years and older, with lived experience of suicidal behaviour that includes thoughts of self-harm or suicide, or previous self-harm behaviour, will be recruited using purposive sampling. Older adults with active suicidal ideation or who had a self-harm presentation within the past 3 months will be excluded. Active suicidal ideation will be assessed by the interviewer when describing the study details to potential participants. Older adults who have significant communication barriers (e.g. hearing or speech impairments that result in the inability to provide informed consent) or a diagnosed cognitive impairment or dementia that could interfere with informed consent or participation will be excluded. Participation from different genders, ethnic groups, geographical areas and age groups of older adults will be sought, and the Lived Experience Panel will support further identification of recruitment strategies should this be required. Older adults will be recruited via relevant charities/organisations and media/social media.

#### Carers

Carers (either family or friends) of older adults with lived experience of suicidal behaviour will be recruited via social media, relevant organisations and support groups targeted for carers. Carers will not be approached directly via older adult participants, nor will older adults be asked to pass on carer contact details.

#### Healthcare professionals

Healthcare professionals with experience supporting individuals with suicidal behaviour will be invited to take part in this study. Purposive sampling will be used to ensure representation from the different healthcare professional groups targeted (general practitioners, suicide crisis assessment nurses, clinical nurse specialists, psychiatrists and other relevant medical professionals), as well as representation from women and men. Recruitment will be conducted via media/social media platforms, word of mouth via the Academic/Clinician Advisory Group, snowball sampling and contacting professional organisations.

#### Data collection

Data collection will be via individual semi-structured interviews. The semi-structured interview approach ensures that key aspects are explored, while also allowing the space for participants to express themselves freely.^35^ Interviews will be either by telephone/online platform or in person, as selected by the participants.

Three separate topic guides will be developed to support data collection, each tailored to one of the participant groups: (a) older adults with lived experience of suicidal behaviour, (b) carers/family supporters and (c) healthcare professionals. The topic guides will be co-developed with the Lived Experience Advisory Group and informed by previous research, including earlier work packages. They will be revised iteratively alongside data collection. The topic guides will cover: (a) the needs of older adults with suicidal behaviour; (b) the barriers and facilitators to providing healthcare support and caregiving; and (c) the perspectives and experiences of older adults, carers and healthcare professionals on existing services and how they could be improved.

‘Information power’^36^ will inform the approximate number of participants to take part in the study. Information power states that data collection in qualitative research should cease when major categories have provided depth and variation, a concept that will be adhered to in all participant groups.^36^ In line with previous studies, we expect information power to be reached within 10–15 interviews from each participant group, but will adhere to the concept and continue data collection until information power is reached.

Interviews will be audio-recorded with consent from participants and field notes will be taken by the researcher to document reflexivity.

#### Analysis

Reflective thematic analysis will be used to analyse the data. Interviews will be transcribed verbatim, anonymised and imported to NVivo 14 Plus for Windows (Lumivero, Denver, Colorado, USA; www.lumivero.com/products/nvivo/) to begin the data analysis process.^37^ Data will be analysed inductively using a data-driven approach, keeping researcher subjectivity central to the analytical process. All transcripts will be coded by the first author, and approximately 40% of the transcripts will be independently open-coded by another member of the research team and the Lived Experience Advisory Group, as a validity check. A comparison of these short and descriptive codes will then follow, until consensus is reached. Should there be any disagreements, a third member of the research team will be involved. Wider categories and subtheme development will follow, involving further interpretation of the data-set and exploring the relationship between codes to generate themes. Initial candidate themes will be generated, which will then be refined and revised into final themes.^37^

The Lived Experience Advisory Group will be involved in the data analysis and reflections of having researchers and members with lived experience in the data analysis process will be central to the data analysis process. Reflexive practice will be an integral part of the analytical process to actively examine assumptions, positions and any tensions between authors. Regular meetings will be held to discuss the data analysis at different stages, including transcripts review, preliminary codes and themes.

### Ethics and consent statements

The authors assert that all procedures contributing to this work comply with the ethical standards of the relevant national and institutional committees on human experimentation and with the Helsinki Declaration of 1975, as revised in 2013. All procedures involving human patients were approved by the Social Research Ethics Committee at University College Cork (Log No: 2025-129).

Written informed consent will be obtained for all participants recruited for the interview study in stage 4. Consent to participate is not applicable to participants in stages 1–3, as they relate to secondary data analysis of already collected data-sets.

### Advisory groups

There are two expert advisory groups that will be involved with this research. The first is the Lived Experience Advisory Group, composed of seven representatives: three older adults with lived experience of suicidal behaviour and four carers/family members of older adults with lived experience of suicidal behaviour. One initial meeting was held in April 2025 with the Lived Experience Advisory Group to discuss the study protocol, and a further five meetings are planned for the duration of this research. The involvement of the panel will include advising the recruitment strategies and data analysis of the qualitative research, interpretation of findings and dissemination of the research.

The second advisory group is the Academic/Clinician Advisory Group, composed of 13 experts in the field of suicide prevention and healthcare who will advise on the methods and conduct of the research as well as facilitate recruitment.

## Discussion

Although there is an increased awareness and understanding of the importance of suicide prevention in older adults, up-to-date national research informed by those with lived experience is needed for improved service provision. This study aims to address several research gaps that will help inform service provision in Ireland. First, using one of the few national self-harm surveillance systems, we will investigate the impact, if any, of the COVID-19 pandemic on hospital-presenting self-harm in older adults, and identify demographic subgroups at increased risk. Second, we will provide a national descriptive profile of older adults dying by suicide in Ireland. Third, complementing the previous study, we will examine the risk of suicide and repeat self-harm in older adults following hospital-presenting self-harm, by using data linkage of two national databases. Finally, using qualitative methods, we will examine the perspectives of older adults, healthcare practitioners and carers, to understand the service needs of older adults with suicidal behaviour to support improvements in clinical care. By gaining an up-to-date national overview of hospital presenting self-harm and probable suicide deaths in older adults, the sociodemographic and clinical profile of this age group will be identified to inform future suicide prevention strategies. Data linkage of two large national databases will add to existing research by establishing a national cohort of individuals with hospital-presenting self-harm and examining their risk of suicide. This research will be informed by two advisory board panels, including a Lived Experience Advisory Panel, which will enhance the relevance and impact of this research. The four study objectives are interrelated and connected to gain a better understanding of suicidal behaviour in later life, and will inform the second part of this research to be led by the authors, which includes co-design of guidelines for suicide prevention of older adults in Ireland.

The proposed study builds on existing national^13,18,24,38,39^ and international research^8,16^ examining suicidal behaviour in older adults. Although nationally, previous research has addressed suicide,^24^ hospital-presenting self-harm^13,24^ and suicidal ideation^14^ in older adults, the risk of suicide following self-harm has not been examined in this age group. Furthermore, several of the mentioned studies report on data that is over a decade old, and up-to-date evidence is required. A study using the NSHRI database was published by several of the authors in 2024,^13^ but this study analysed hospital-presenting self-harm rates from 2007 to 2019, leaving a clear gap in relation to more recent data. The increased risk of suicide following hospital-presenting self-harm has been identified by Griffin and collaborators;^18^ however, further examination of the identified high-risk group of older adults is needed. The service needs of older adults who self-harm have not been examined, which are key to inform improved health service provision.

### Strengths and limitations

Strengths of this research are its mixed-methods study design with multiple data sources, including two national databases (NSHRI and IPSDS), which not only makes the research nationally representative, but also may allow for international comparison. The fourth stage of this research will provide critical in-depth information from three different participant groups to improve service provision for older adults with suicidal behaviour. Furthermore, two advisory boards inform this research, including a Lived Experience Advisory Group and Academic/Clinician Advisory Group, which will enhance the conduct and quality of the research.

The NSHRI is one of the few national surveillance systems worldwide that provide national self-harm data. The NSHRI uses standardised definitions and inclusion criteria, with trained data registration officers involved in data collection. This unique data-set will allow us to examine national self-harm rates across older adults living in Ireland. The NSHRI includes important variables such as provision of an assessment, referral outcome and alcohol involvement, among others. However, it does not include other relevant data for understanding suicidal behaviour, such as motivations for self-harm, levels of suicidal intent, previous psychiatric diagnoses, ethnicity and physical health conditions, among others. We will be unable to account for repeated individual presentations in the calculation of rates of hospital self-harm presentations, as we can only consider one self-harm presentation per person per year. Considering the number of individuals who repeat self-harm within a year, this is a limitation of the study design. Furthermore, when estimating the effects of the COVID-19 pandemic on self-harm rates, it is important to acknowledge the potential influence of other concurrent or overlapping societal events, such as economic challenges or health system pressures, which may also have contributed to observed trends. This limits the ability to isolate the specific impact of the pandemic. Finally, the NSHRI captures only hospital-presenting self-harm, meaning only a small percentage of those who self-harm is represented, given that a large proportion of self-harm episodes occur within community settings.^39^

As this study is based on observational data, causal relationships cannot be established. Findings on the identification of adverse life events can only suggest associations rather than direct causation. Variables identified as being associated with suicide may not necessarily have contributed to the outcome. The IPSDS is a rich national study that has national coverage of suicide deaths from 2015 to 2020. A strength of the IPSDS is that it includes both coroner-determined suicides and research-determined suicides, following rigorous investigations.^32^ However, variability in how coroners conduct their investigations may affect the information recorded in their files, therefore the missingness level of specific IPSDS variables should be treated with caution.^40^ Furthermore, although the IPSDS is a rich dataset, key details such as ethnicity, deprivation levels and migrant status are not captured.

Although data linkage of two national databases will allow for enhanced understanding of suicide risk, given that there are no patient identifiers in Ireland, we will use deterministic record linkage. This methodology, as described previously, requires matching variables and as such, coding errors may occur. However, data from both sources are recorded in a systematic way by trained researchers, minimising the opportunity for coding errors.^18^

Suicidal behaviour is a complex phenomenon, and qualitative data can often aid in further understanding this complexity. Interviews with three relevant groups (healthcare practitioners, carers and older adults with lived experience of suicidal behaviour) will offer important insights on suicide prevention in older adults. It is important to highlight qualitative studies are mostly not generalisable, but they can lead to rich insights that inform research and practice and are transferable across similar contexts. We will aim to recruit a diverse group of participants, but qualitative research can often lack representativeness of minority groups. However, as outlined in the Method section, we will continue data collection until saturation of data has been reached, and have diverse recruitment strategies informed by the two advisory groups to enhance recruitment and participation.

To summarise, this study protocol describes a four-stage, mixed-methods research proposal that will examine and identify factors associated to self-harm and suicide in older adults living in Ireland, using two national databases (including data linkage), triangulated through qualitative research. This research will be informed by two advisory groups (Lived Experience and Academic/Clinician). This research will address national evidence gaps to better understand and care for suicidal behaviour in older adults. Although this protocol is developed using Irish data sources, the methodological approach is transferable to other countries with similar population-based data-sets. Adapting this framework elsewhere would support wider international comparisons and inform service provision in diverse health systems.

## Data Availability

Data availability is not applicable to this article as no new data were created or analysed in this study.
